# Superradiance and Broadband Emission Driving Fast Electron Dephasing in Open Quantum Systems

**DOI:** 10.1002/advs.202522729

**Published:** 2026-03-10

**Authors:** Gimin Bae, Youngjae Kim, Jae Dong Lee

**Affiliations:** ^1^ Department of Physics and Chemistry DGIST Daegu Republic of Korea; ^2^ School of Physics KIAS Seoul Republic of Korea

**Keywords:** broadband emission, correlated electron systems, Dicke superradiance, high‐harmonic generation, Lindblad equation

## Abstract

The physical origin of the ultrashort dephasing time *T*_2_ (≈ O(1) fs), persistently addressed in the solid‐state high‐harmonic generation (HHG), remains an unresolved and challenging issue because the electron scattering at such a short timescale within solids is hardly identified. Here we investigate HHG of the 1D Hubbard model in the dissipative quantum environment within a frame of the Lindblad equation. In the study, in the limit of a small interatomic distance, we make the first verification of the solid‐state Dicke superradiance, the coherent stimulated emission triggered by the spontaneous emission, as well as the broadband emission mimicking the blackbody radiation due to the electron scattering in each harmonic multiple. Further, we find a strong destructive interference between the Dicke superradiance and the broadband emission, which makes a scale down of the effective electron scattering time and leads to just few‐femtosecond dephasing time *T*_2_. This finding explains a long‐standing problem of the ultrafast femtosecond electron dephasing in HHG. The present study could also serve as a pertinent platform for understanding the nonequilibrium dissipative dynamics of correlated electron systems.

## Graphical abstract

We uncover the physical origin of ultrafast electron dephasing in solid‐state high‐harmonic generation by simulating the Lindblad equation for the dissipative Hubbard model. Coexistence of Dicke superradiance and broadband emission is revealed, whose destructive interference shortens the effective scattering time and provides a unified picture of electron dephasing in solids.

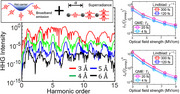

## 1 Introduction

Since an observation of the high‐harmonic generation (HHG) in solids, which is observed even with a few‐microjoule optical pumping energy, HHG has been in the spotlight for the application to the coherent light conversion and the ultrafast dynamics as well as the nonequilibrium phenomena, and also for the spectroscopic availability to explore the fundamental physics of materials [1]. This has been demonstrated by extracting the material symmetry [2, 3], the electronic structure [4, 5, 6, 7], the transition dipole matrix [8], and the Berry curvature [9, 10]. Probably the most powerful advantage of the HHG spectroscopy is to enable an identification of time‐resolved excited dynamics. The quantum master equation (QME) considers an exponential decay due to the spontaneous emission in the excited state of a two‐level tight‐binding (TB) system [11, 12], which allows a direct comparison between experiment and theory [13, 14]. This reveals that dephasing dynamics are crucial in the HHG process of condensed matter systems, and it serves as a powerful method for determining the dephasing time *T*_2_ [15]. The dephasing time *T*_2_ is estimated to be just a few femtoseconds, being so short that it is difficult to find comparable relevant time scales in the system. A recent study has suggested that the light propagation may induce a drastic dephasing [16]. However, the mechanism requires a sufficient propagation length (≈ O(100) µm), which is not attainable in more recently reported 2D materials (e.g., MoS_2_ [14] and graphene [15]). Despite an excellent agreement between theoretical calculations and experiments with an assumption of such a few‐femtosecond *T*_2_, the physical origin of the extremely short *T*_2_ is a long‐standing puzzle in the solid‐state HHG [17, 18, 19].

Various studies analyze the HHG mechanism from a microscopic perspective, such as the electron‐electron interaction [20], the electron‐phonon interaction [21], and the material topology [22]. These studies employ the time‐dependent density functional theory, considering the electron dynamics, incorporating the electron correlation via the Hubbard *U* [20] and the coherent phonon [21], and the time‐dependent Schrödinger equation (TDSE) [23] or the semiconductor Bloch equation. In particular, the many‐body dynamics adopting the 1D Hubbard model analyze HHG spectra with respect to the Hubbard *U* and address the insulator‐metal transition with the few‐femtosecond accuracy [23]. The dynamical study copes with the unitary evolution of TDSE of the 1D Hubbard model, but a direct comparison with the phenomenological QME accounting for the electron dephasing in the density operator matrix seems to be ambiguous. Recently, as an important feature of the electron‐electron scattering, the broadband emission in addition to odd‐order harmonics is observed in 2H‐NbSe_2_ [24]. The broadband emission is generated from a kind of hot carriers, which is found to mimic the blackbody radiation at an effective temperature. Besides, an interplay between the electron scattering and the optical driving enhances the nonperturbative nature of high harmonics.

To draw clearer scientific insight into the electron dephasing in correlated electron systems, one may need to properly describe the dissipative dynamics of correlated electrons and go to the Hubbard chain coupled to the dissipative quantum environment instead of the isolated one [25]. In this case, another important radiative process due to the electron‐environment scattering as well as the electron‐electron scattering, called the Dicke superradiance, may occur [26, 27, 28, 29]. The Dicke superradiance is the light emission steered by a collective deexcitation of atoms much stronger than the emission of independent atoms, i.e., the coherent stimulated radiation triggered by the spontaneous emission, especially in the limit that the interatomic distance goes to zero. In correlated electron systems, in the sense, the Dicke superradiance may be a key essence to determine the dephasing dynamics of electrons together with the broadband emission.

In this paper, we utilize the Lindblad equation combined with 1D Hubbard model and investigate the electron dynamics of HHG in the dissipative open quantum system. Especially at a weak or moderate correlation, i.e., *U*/*t* mainly between 1 and 3, as the Lindblad coupling, regarded as the electron‐environment scattering, turns on, each harmonic becomes to be suppressed systematically due to a destructive interference between the broadband emission and the Dicke superradiance. The broadband emission is structureless due to hot carriers produced by the electron‐electron scattering. In particular, the Dicke superradiance could be separated from the whole HHG spectra and found to drastically increase as the interatomic distance decreases, which is in fact, verified by indicating the *N* ^2^‐scaling of the intensity, where *N* is the number of atoms a unit length. Furthermore, such a destructive interference is also found to make a scale down of the electron scattering time, eventually leading to the few‐femtosecond time scale of *T*_2_, which has been an open problem of HHG studies based on the phenomenological QME [13, 14, 15, 16, 17, 18, 19]. This finding could offer a microscopic explanation for the problem. The present work also paves the way for unveiling the highly nonequilibrium dynamics of correlated electron systems with dissipations.

## 2 Results and Discussion

### 2.1 Lindblad Equation and Light‐Emitting Processes

A model of atoms in an optical lattice could be adopted as it is for correlated electrons in a crystalline lattice. For the many‐body dissipative dynamics of fermionic atoms trapped in an optical lattice, the master equation for the density operator ρ(τ) could be derived through the effective non‐Hermitian Hamiltonian *H*_eff_ [25]

(1) $$\frac{\partial }{\partial \tau }\rho \;\left(\tau \right)=-i\left[H_{\mathrm{eff}}\;\rho \left(\tau \right)-\rho \left(\tau \right)H_{\mathrm{eff}}^{†}\right]+\zeta \rho \left(\tau \right)$$

where *H*_eff_ is given by $H_{\mathrm{eff}}=H_{0}+H_{\mathrm{eff}}^{\mathrm{int}}+H_{\mathrm{eff}}^{\mathrm{light}}$ and ζρ(τ) is the recycling term. *H*_0_ is the Hamiltonian for noninteracting atoms in an optical lattice, $H_{\mathrm{eff}}^{\mathrm{int}}$ the collisional interaction between atoms, and $H_{\mathrm{eff}}^{\mathrm{light}}$ a collective two‐atom excitation and deexcitation incorporating the Wigner‐Weisskopf spontaneous emissions [11, 12]. ζρ(τ) controls an action of the environment and enables to preserve the trace of the density operator. In the present case of the 1D Hubbard model (see Methods), we have $H_{\mathrm{eff}}^{\mathrm{int}}=H_{U}$, $H_{\mathrm{eff}}^{\mathrm{light}}=i\frac{\gamma }{2}\sum_{i}L_{i}^{†}L_{i}$, and $\zeta \rho \;\left(\tau \right)=\gamma \sum_{i}L_{i}\rho \left(\tau \right)L_{i}^{†}$. *L_i_* is the Lindblad operator at each site and γ the Lindblad coupling constant denoting the electron‐environment scattering strength. In particular, it is noted that $i\frac{\gamma }{2}\sum_{i}L_{i}^{†}L_{i}$, which depicts the collective deexcitation, should have basically the same physical origin with *H_U_*, that is, the Coulomb correlation between electrons. Under the optical pump pulse *A*_pump_(τ) belonging to the mid infrared range in the present study, the nonlinear current leading to HHG can be evaluated from the density operator of the Lindblad master equation.

**FIGURE 1:**
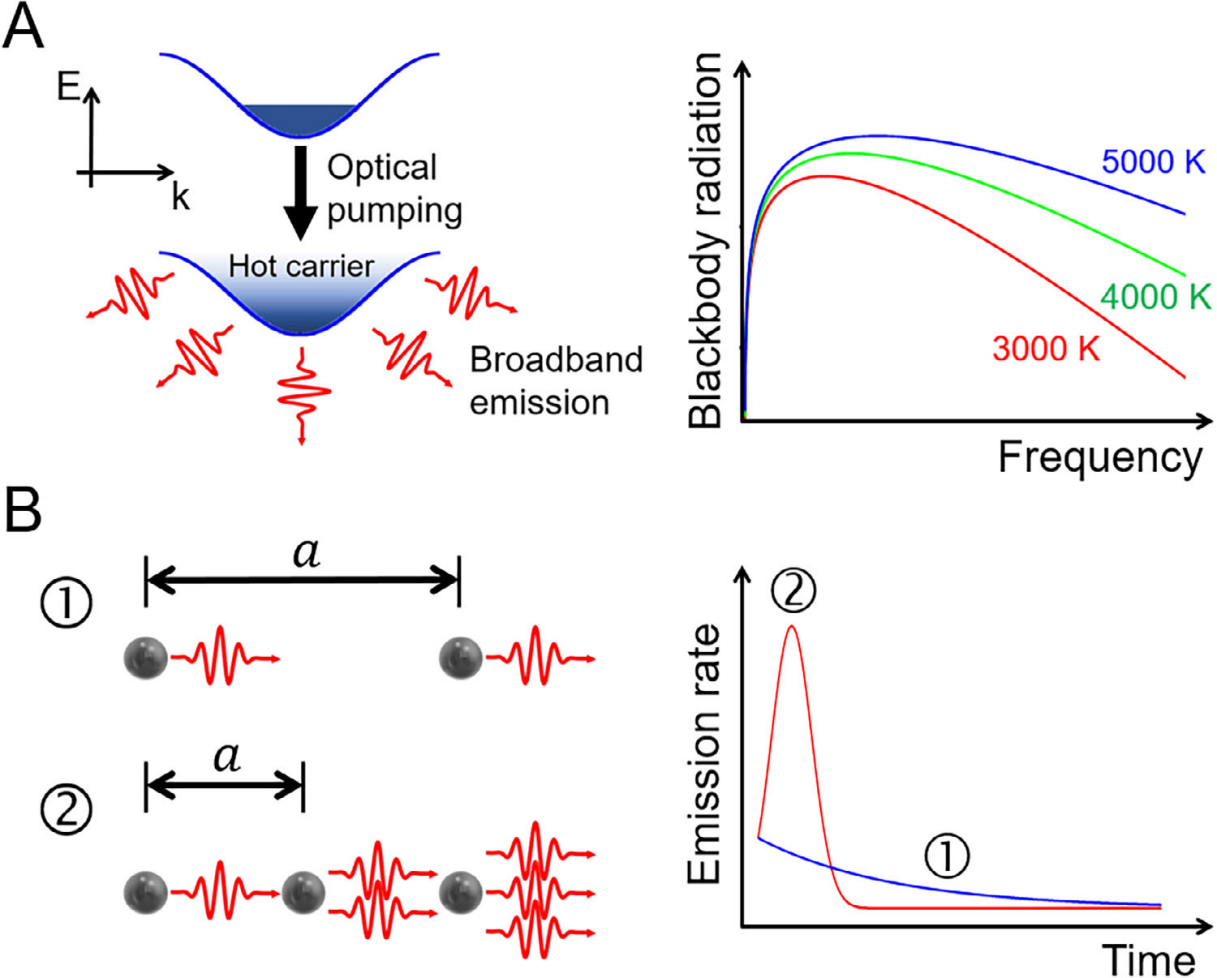
Light‐emitting processes in a correlated electron system. (A) Broadband emission generated by hot carriers due to the electron‐electron scattering, which is known to mimic the blackbody radiation (note a logarithm‐scaled sketch) at effective temperatures. (B) Spontaneous emission of independent atoms (denoted by ①) and the Dicke superradiance (denoted by ②) due to a collective deexcitation of atoms in the limit of a small interatomic distance *a*, i.e., a→0 (note that, for the display, *a* is illustrated intentionally longer compared to the wavelength).

Dynamical evolution of the electron system under the master equation would be significantly different between cases without and with the electron‐electron scattering. Without the electron scattering, although electrons in the ground state would be accelerated and displaced by the optical pumping, electrons would be back to the initial ground state after the pumping. In contrast, with the electron scattering, evolving the initial ground state shows that accelerated and displaced electrons would be fallen to a high‐temperature‐like electron distribution, say scattered hot carriers (i.e., in order to distinguish from thermal hot carriers). Such scattered hot carriers would make the thermal‐like radiation and contribute the structureless broadband emission to HHG, which is illustrated in Figure 1A. The broadband emission is reported to mimic the blackbody radiation formula at a given effective temperature [24]. On the other hand, if the deexcitation dynamics due to the spontaneous emission, as described by a term of $H_{\mathrm{eff}}^{\mathrm{light}}$ of Equation (1), are involved, the Dicke superradiance of Figure 1B would be enabled in appropriate limits. When an electron is excited by the optical pumping, a spontaneous emission process occurs. A neighboring electron then undergoes a stimulated emission. This collective emission process in the form of a burst of light with a very short time scale (Figure 1B) would be active when the interatomic distance *a* is vanishingly small, i.e., a→0 [29]. That is, the interatomic distance should be much shorter than the wavelength corresponding to an energy difference between levels. The condition could be satisfied without much difficulty for ordinary semiconductors such as graphene and transition metal dichalcogenides [30, 31]. The superradiance much faster and stronger than the emission of independent atoms, would be actually expected in HHG of condensed matter systems.

### 2.2 Broadband Emission and Dicke Superradiance

**FIGURE 2:**
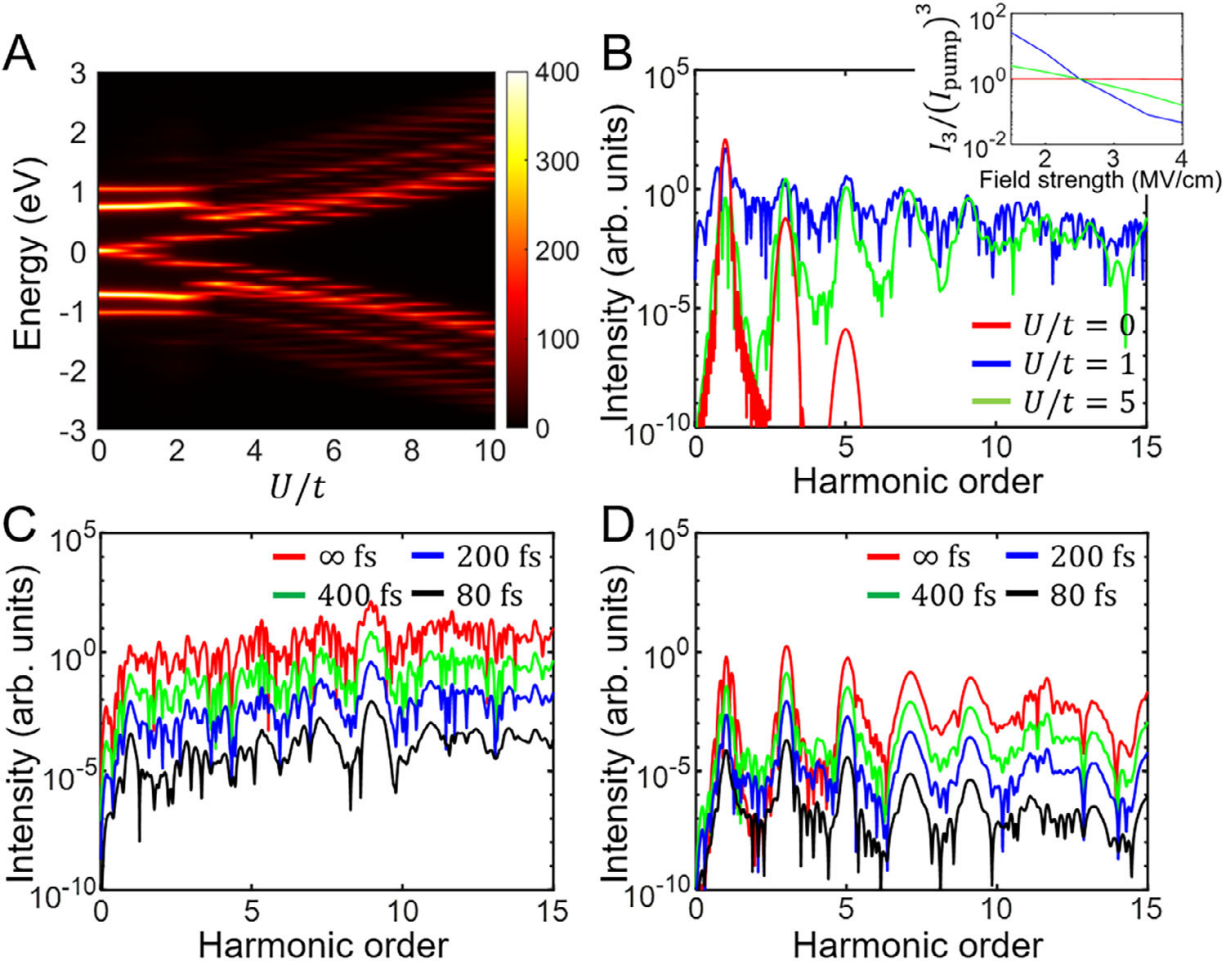
HHG spectra of the 1D Hubbard model with the Lindblad equation. (A) Spectral function of the 1D Hubbard model with respect to *U*/*t*. (B) HHG spectra at γ^−1^ = ∞ fs for *U*/*t* = 0, 1, and 5 with the optical field strength of 3.5 MV/cm. The broadband emission is observed at *U*/*t* = 1. The inset gives a ratio of the third‐order harmonic intensity and the cube of the optical pump intensity, i.e., $I_{3}/I_{\mathrm{pump}}^{3}$, with respect to the optical field strength. (C,D) HHG spectra at *U*/*t* = 5 for γ^−1^ = ∞, 400, 200, and 80 fs with the optical field strengths of 10 MV/cm (C) and 3 MV/cm (D). For a better shaped demonstration, spectra are vertically offset by successively multiplying a factor of 10^−1^ in (C) and (D).

In a treatment of the 1D Hubbard model *H* (= *H*_0_ + *H_U_*), we assume a half‐filling case with *N_S_* = 8 unless mentioned otherwise, where *N_S_* is the number of sites. Figure 2A provides the spectral function *A*(ω) of the 1D Hubbard model (see Methods). The spectral function is well known to be determined entirely by a value of *U*/*t*, where *U* is the on‐site Coulomb repulsion and *t* the hopping parameter. According to Figure 2A, the Hubbard system would be metallic at U/t≲1, whereas the system becomes insulating at *U*/*t* ≫ 1. Further, *U*/*t* = 1 − 3 would be mostly on the threshold where the insulator‐metal transition occurs. Evolving the many‐body ground state with the Lindblad master equation incorporating an additional scattering with the environment, we evaluate the HHG spectra (see Methods). In Figure 2B, with the vanishing Lindblad coupling, i.e., γ^−1^ = ∞, HHG spectra are delivered at *U*/*t* = 0, 1, and 5. For *U*/*t* = 0, only odd‐order harmonics are expected by the symmetry of the Hubbard model [32]. But an inclusion of the electron scattering gets to induce the broadband emission regardless of odd or even order. Actually, odd‐order harmonic structures fairly clear at *U*/*t* = 0 and 5 tend to be veiled by the structureless broadband emission at *U*/*t* = 1, which could be actually fitted with the even‐order harmonics by the blackbody radiation formula with an effective temperature *T* = 3800 K (Figure S1). This is consistent with that the broadband emission is substantial in the correlated metallic phase [24], i.e., at a weakly or moderately correlated regime like 1≲U/t≲3. It is also shown in the inset of Figure 2B that interplays between scattering and pumping processes intensify the nonperturbative behaviors of harmonics, consistently with the recent experiment [24]. In Figure 2C,D, HHG spectra at a strong correlation of *U*/*t* = 5 are demonstrated for the optical field strengths (i.e., $E_{\mathrm{pump}}^{0}$) of 10 MV/cm and 3 MV/cm, respectively, with respect to various Lindblad coupling constants given by their inverse values, i.e., γ^−1^ = ∞, 400, 200, and 80 fs. γ^−1^ may in fact denote the corresponding electron scattering time. Harmonic structures are revealed to be clearer at a weaker field strength, which is consistent with diabatic processes for nonperturbative mechanisms of HHG [33]. Going to much higher‐harmonic order, the overall spectral peak resides near the doublon excitation energy, i.e., *U*/ω_pump_, and allowed excitations are shown to have energies between Δ and Δ + 8*t* [23, 34], where Δ is an optical gap (Figure S3). This may define the lower and upper frequencies for the HHG emission at a given value of *U*/*t* (≫1).

**FIGURE 3:**
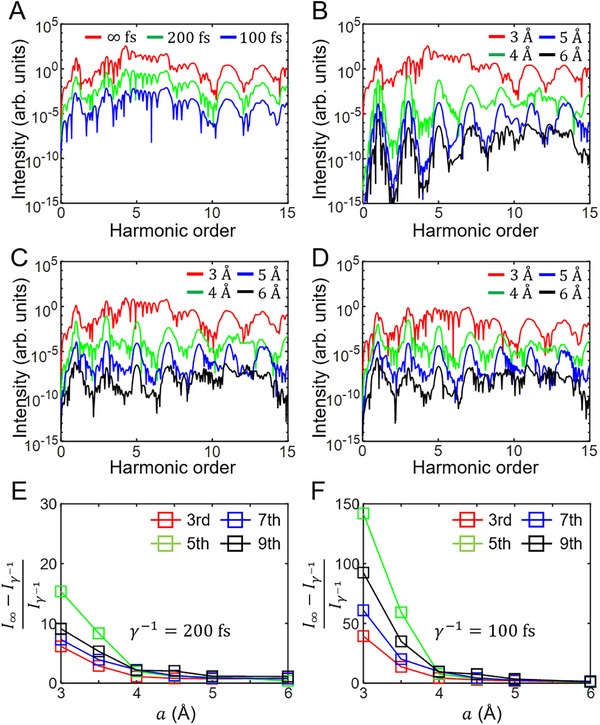
HHG spectra: Dicke superradiance and broadband emission. (A) HHG spectra at *U* = 2.6 eV and *a* = 3 Å (assuming *t* = 0.92 eV) for γ^−1^ = ∞, 200, 100 fs with the optical field strength of 3 MV/cm. (B‐D) HHG spectra at *U* = 2.6 eV and γ^−1^ = ∞ fs (B), γ^−1^ = 200 fs (C), and γ^−1^ = 100 fs (D) for *a* = 3, 4, 5, and 6 Å. For a better shaped demonstration, spectra are vertically offset in (A‐D) in the same fashion as Figure 2C,D. (E, F) Normalized differences between harmonic intensities at γ^−1^ = ∞ and γ^−1^ = 200 fs (E) and at γ^−1^ = ∞ and γ^−1^ = 100 fs (F) for the third, fifth, seventh, and ninth order harmonics with respect to *a*. Normalized difference, i.e., $\left(I_{\infty }-I_{\gamma^{-1}}\right)/I_{\gamma^{-1}}$, corresponds to an approximation of the Dicke superradiance in the limit of small *a*.

**FIGURE 4:**
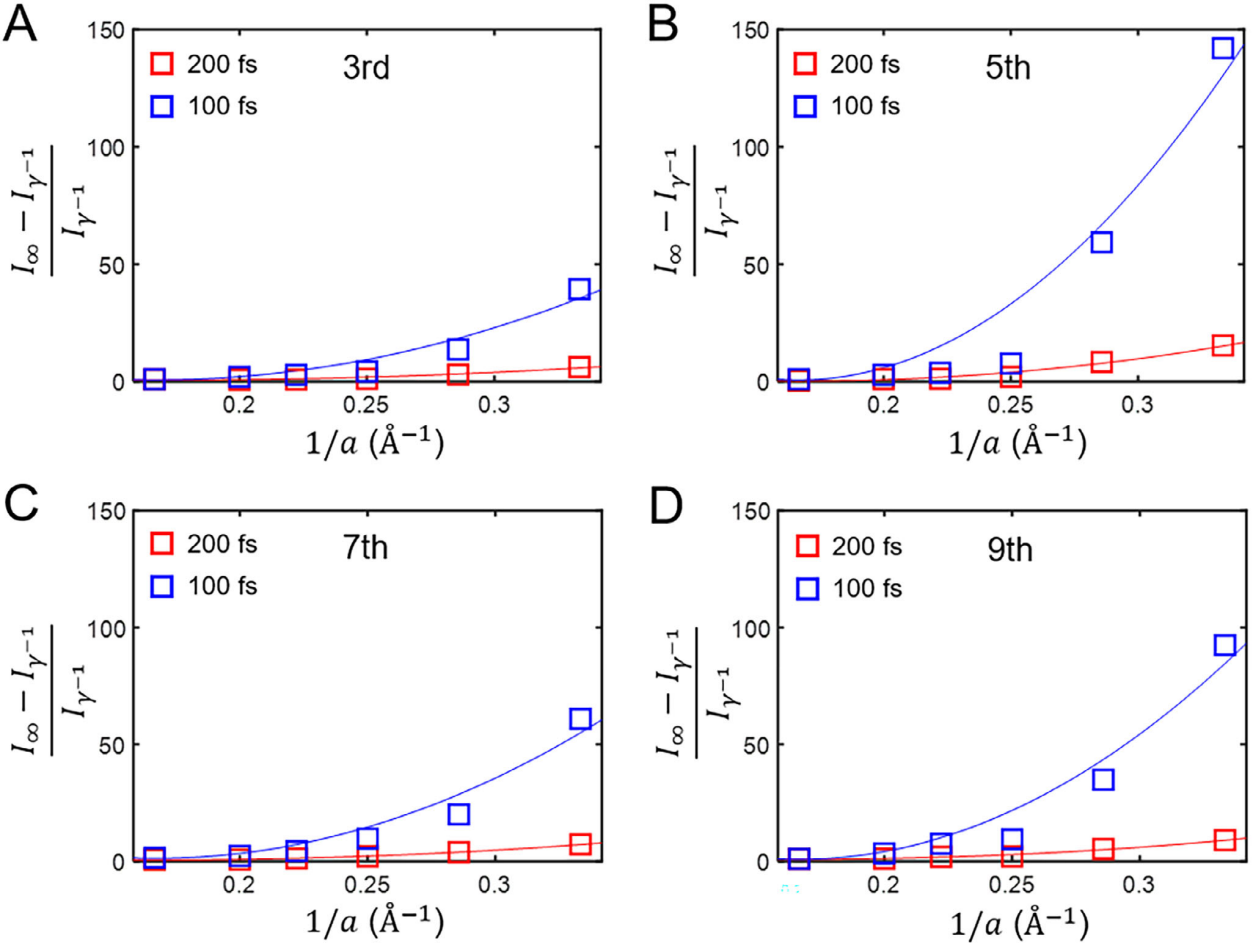
*N* ^2^‐scaling of Dicke superradiance with *N* (∝1/*a*) the number of atoms a unit length. (A‐D) Normalized differences between harmonic intensities at γ^−1^ = ∞ and γ^−1^ = 200 fs (red) and at γ^−1^ = ∞ and γ^−1^ = 100 fs (blue) for the third (A), fifth (B), seventh (C), and ninth (D) order harmonics with respect to 1/*a*. Normalized difference, i.e., $\left(I_{\infty }-I_{\gamma^{-1}}\right)/I_{\gamma^{-1}}$, corresponds to an approximation of the Dicke superradiance in the limit of small *a*. The same parameters are adopted as in Figure 3. The lines represent quadratic fits to the data.

In Figure 3A, HHG spectra are evaluated at fixed values of *U* = 2.6 eV and *t* = 0.92 eV (i.e., *U*/*t* = 2.8) for γ^−1^ = ∞, 200, and 100 fs. In the figure, for the hopping parameter of t=0.92eV, the interatomic distance a=3Å is assumed. In Figure 3B‐D, HHG spectra are further evaluated for γ^−1^ = ∞, 200, and 100 fs, respectively, as the interatomic distance *a* is explicitly elongated from 3Å to 6Å in the system adopted for Figure 3A. A relation between the hopping parameter and the interatomic distance is simply assumed to be *t*∝1/*a* ^2^. As *a* increases from 3Å to 6Å, *U*/*t* accordingly increases following ≈ *a* ^2^ and odd‐order harmonic structures become more and more conspicuous in each figure. Further, it should be noticed that the absolute intensities of harmonic spectra monotonically decrease as γ^−1^ decreases order by order at all values of the given interatomic distances. For the third, fifth, seventh, and ninth order harmonics, the normalized differences between those absolute intensities at γ^−1^ = ∞ and γ^−1^ = 200 fs are investigated in Figure 3E, and similarly, the differences between at γ^−1^ = ∞ and γ^−1^ = 100 fs in Figure 3F with respect to the interatomic distance *a*, which are discovered to dramatically increase as *a* decreases. Moreover, the normalized differences at γ^−1^ = 100 fs show a more prominent increase than those at γ^−1^ = 200 fs. Most of all, as displayed in Figure 4, all the normalized differences for the third, fifth, seventh, and ninth order harmonics, regardless of the value of γ^−1^ are found approximately to be ∝ (1/*a*)^2^, i.e., ∝*N* ^2^, where *N* is the number of atoms a unit length. Of course, the proportionality coefficients are much larger at γ^−1^ = 100 fs than at γ^−1^ = 200 fs. This is important evidence of the Dicke superradiance that the intensity becomes proportional to the square of the number of radiators [26]. Such an observation lets us conclude that the broadband emission and the Dicke superradiance destructively interfere with each other at finite values of γ^−1^ and the normalized difference, i.e., $\left(I_{\infty }-I_{\gamma^{-1}}\right)/I_{\gamma^{-1}}$ roughly corresponds to the Dicke superradiance. As a matter of fact, it can be shown that $\left(I_{\infty }-I_{\gamma^{-1}}\right)/I_{\gamma^{-1}}$ approximates the Dicke superradiance in such destructive interference in the limit of a small interatomic distance (Supporting Information). Moreover, the Dicke superradiance is strongly enhanced just at weakly or moderately correlated regime, i.e., 1≲U/t≲3 (Figure S4), similar to a case of the broadband emission as discussed in Figure 2B. This implies that, as for the occurrence condition, the broadband emission and the Dicke superradiance are in fact more or less overlapped, in which the two pathways for the radiation could severely interfere with each other in a destructive fashion. A physical origin of the destructive interference would be possibly the phase difference in the pathways, that is, the electron‐environment scattering is incorporated in the Dicke superradiance, whereas not in the broadband emission.

In correlated systems described by the Hubbard model, HHG can be naturally interpreted in terms of doublon‐holon quasiparticle dynamics. In this framework, the HHG process can be mapped onto a three‐step picture: (i) the creation of doublon‐holon pairs by the pump field, (ii) the propagation of these quasiparticles through the lattice, and (iii) the recombination of doublon‐holon pairs with the emission of photons [35]. Within this quasiparticle‐based description, the Dicke superradiance enters as an additional radiative channel associated with the recombination step. Unlike conventional closed‐system approaches, the inclusion of a dissipative quantum environment enables collective deexcitation of doublon‐holon pairs, leading to an enhanced and phase‐coherent emission of radiation, characteristics of the Dicke superradiance. For this perspective, for understanding of correlated systems in an open environment, a consideration of the Dicke superradiance should be inevitable. It may be also worthy of commenting that, regardless of a detailed form of the spin‐independent Lindblad operator, virtual double occupations decohere due to the spontaneous emission, and in a steady state, the population is distributed in the single‐occupancy states and multiple double‐occupancy states (Figure S5). Hence, discussions concerning the broadband emission and the Dicke superradiance are found to be highly robust at least in a qualitative respect (Figure S6). Lastly, it would be noticeable that the interatomic distance decreases, and a control over coherent and collective quantum effects among emitting sites becomes accessible. This would offer a deeper insight into the way toward quantum optics and quantum information technology [36, 37, 38, 39].

### 2.3 Few‐Femtosecond Dephasing Time

**FIGURE 5:**
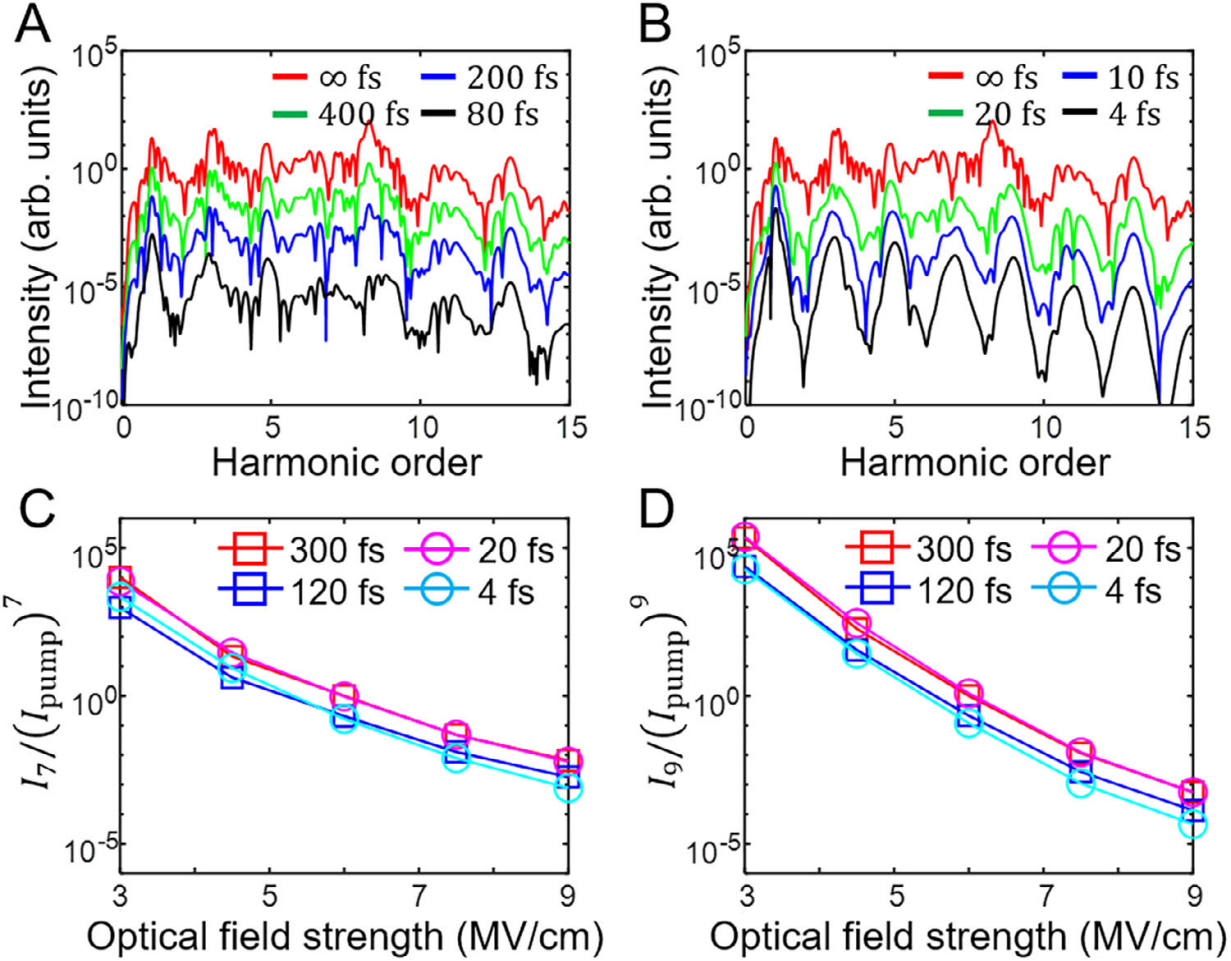
*T*_2_ from the HHG spectra of the 1D Hubbard model. (A) HHG spectra obtained by the Lindblad equation for the 1D Hubbard model (*N_S_* = 6) at *U*/*t* = 2 for γ^−1^ = ∞, 400, 200, and 80 fs with the optical field strength of 3 MV/cm. (B) HHG spectra obtained by an ad hoc extension of QME for *T*_2_ = ∞, 20, 10, and 4 fs. The same parameters are adopted as in (A). For a better shaped demonstration, spectra are vertically offset in (A) and (B) in a same fashion as Figure 2C,D. (C) A ratio of the seventh order harmonic intensities and the seventh of the optical pump intensity, i.e., $I_{7}/I_{\mathrm{pump}}^{7}$ with respect to the optical field strength. (D) A ratio of the ninth order harmonic intensities and the ninth of the optical pump intensity, i.e., $I_{9}/I_{\mathrm{pump}}^{9}$ with respect to the optical field strength. Empty squares (annotated as values of γ^−1^) represent the calculation results of the Lindblad equation, whereas empty circles (annotated as values of *T*_2_) those of QME.

It has been recently reported that the dephasing dynamics are crucially important in the HHG process of the 2D semiconductors [13, 14, 15], where experimental and theoretical analyses are attempted for the effective two‐level TB model with QME employing the 2 × 2 density operator matrix. One interesting point addressed in the reports is the dephasing time *T*_2_ of a few femtoseconds, which may be in fact so short that it looks difficult to find the relevant time scale in the corresponding semiconducting systems. Now it would be interesting to extract *T*_2_ from our results of the Hubbard model evolution with the Lindblad master equation, even if *T*_2_ is basically a phenomenological parameter not given a priori. For this purpose, we suggest an ad hoc extension of QME with the dimension of the Hilbert space managing the Hubbard model, with all the off‐diagonal terms of the density operator matrix replaced by a single relaxation channel with a parameter *T*_2_ (see Methods). Under a fixed value of *U*/*t* (= 2) and the identical optical conditions, Figure 5A supplies the HHG spectra obtained by the Lindblad equation at γ^−1^ = ∞, 400, 200, and 80 fs, whereas Figure 5B shows the HHG spectra by QME at *T*_2_ = ∞, 20, 10, and 4 fs. To be quantitative, we make a comparison between harmonic intensities attained from the two approaches for the seventh and ninth orders in Figure 5C,D, respectively. For both the seventh and ninth orders, the results of the Lindblad equations with γ^−1^ = 300 fs (120 fs) are found to reasonably reproduce those of QME with *T*_2_ = 20 fs (4 fs). We indicate that a scale down of the physical scattering time γ^−1^ would result in the few‐femtosecond time scale of *T*_2_, i.e., *T*_2_ ≪ γ^−1^. The origin of the scale down should be a strong destructive interference between the broadband emission and the Dicke superradiance in a weakly or moderately correlated regime as discussed with Figures 3 and 4.

### 2.4 Experimental Observability and Extensions

It is interesting and important to experimentally distinguish between the two phenomena, i.e., the Dicke superradiance and the broadband emission. For this purpose, we propose the following two approaches. First, the interatomic distance *a* can be systematically tuned by applying strain to the sample [40]. When the interatomic distance is modified, the intensity scaling with respect to the interatomic distance is different between the Dicke superradiance and the broadband emission. The intensity of superradiance scales as *N* ^2^ (N∼1/a), whereas that of the broadband emission as *N*. The difference in the intensity scaling would enable to isolate superradiance from the HHG spectra and allow an experimental distinction between the two emission processes. Second, the phase‐resolved spectroscopy could be employed. Because superradiance involves the explosive emission of phase‐coherent photons, one expects a strong emission concentrated within a very narrow time‐domain window. Thus, by tacking the phase of the emitted light in the time domain, for instance, in a time‐resolved HHG experiment [41], superradiance could be experimentally distinguished from the broadband emission.

Next, we need to discuss realistic pathways for a rigorous validation of the present model using experimental data and ab initio calculations. For the monolayer MoS_2_, the hopping parameter is estimated to be 1 − 2 eV [42], while the Hubbard correlation parameter *U* is reported to be approximately 2 − 2.3 eV [43]. This leads to a value of *U*/*t* = 1 − 2. That is, MoS_2_ is a weakly or moderately correlated material, definitely belonging to the parameter regime intensively explored in the present study, which indicates that the Hubbard model could be meaningfully calibrated to both experimental and ab initio data, and at the same time suggests that the proposed mechanism for the ultrashort *T*_2_ would be applicable to 2D semiconductors. Actually, the physical scattering time γ^−1^ with the value of a few hundred femtoseconds, being associated with the spontaneous emission process, reasonably reproduces the ultrafast *T*_2_ (≈O(1) fs) which is obtained within the framework of the quantum master equation (QME). This is consistent with recent experimental observation of HHG in MoS_2_ [14]. For future insight, more systematic calibration of the Hubbard model to realistic data for a broad class of correlated materials would be essential for extending the applicability of the proposed mechanism and simultaneously for understanding the limitations of the mechanism.

## 3 Conclusion

We have evolved the electron dynamics of the 1D Hubbard model employing the Lindblad master equation and provided a new scientific insight to the HHG process in view of the dissipative open quantum system. At a weak or moderate electron correlation, i.e., 1≲U/t≲3, it is found that the structureless broadband emission due to hot carriers dispersed by the electron‐electron scattering and, in the limit of a small interatomic distance, the Dicke superradiance driven by the electron‐environment scattering as well as the electron‐electron scattering prevail in the HHG spectra. The broadband emission arising from the electron‐electron scattering has been directly observed in recent HHG experiments on the metallic phase of 2H‐NbSe_2_, where electron‐electron interactions are known to play a crucial role [24]. Here we could have explored a destructive interference between the broadband emission and the Dicke superradiance and, in particular, also made the first verification of the Dicke superradiance in the solid‐state HHG, whose intensity is demonstrated to rapidly increase as the interatomic distance decreases and simultaneously follow the *N* ^2^‐scaling with *N* the number of emitters a unit length. Such a destructive interference is found to make a scale down of the electron scattering time and eventually lead to the few‐femtosecond time scale of *T*_2_, which has been persistently addressed in HHG studies of 2D semiconductors, for instance, graphene [15] and the monolayer MoS_2_ [14]. The present work could explain the extremely fast electron dephasing on a microscopic foundation and should be a milestone for the dissipative many‐body electron dynamics of correlated electron systems, advancing the next generation of quantum technologies. Finally, revealing the emergent properties of the Dicke superradiance also offers insights into coherent and collective quantum effects induced through the many‐body electron dynamics.

## 4 Methods

### 4.1 Calculation of HHG with the Lindblad Equation

We consider the half‐filled 1D Hubbard model [23],

(2) $$H=H_{0}+H_{U}=-t\sum_{\sigma }\sum_{i}^{N_{S}}\left(c_{i\sigma }^{†}c_{i+1\sigma }+H.c.\right)+U\sum_{i}^{N_{S}}c_{i↑}^{†}c_{i↑}c_{i↓}^{†}c_{i↓}$$

where *t* is the hopping parameter and *U* the on‐site Coulomb repulsion. *N_S_* is the system size, i.e., the number of sites. $c_{i\sigma }^{†}\;\left(c_{i\sigma }\right)$ is the electron operator with spin σ at the site *i*. We apply the periodic boundary condition $c_{i\sigma }=c_{i+N_{S}\sigma }$. The spectral function *A*(ω) of the 1D Hubbard model is given through the Lehmann representation

(3) Aω=−Im∑iσ[Ψ0ciσ†1ω−E0+H+i0+ciσΨ0+Ψ0ciσ1ω+E0−H+i0+ciσ†Ψ0]

*E*_0_ is the ground state energy and |Ψ_0_〉 the many‐body ground state wavefunction. Under the optical pump field *E*_pump_ (τ) = − ∂*A*_pump_(τ)/∂τ, the induced nonlinear current *J*(τ) would be calculated by the Lindblad equation [11],

(4) $$\frac{\partial }{\partial \tau }\rho \;\left(\tau \right)=-i\left[H\left(\tau \right),\;\rho \left(\tau \right)\right]+\gamma \sum_{i}^{N_{S}}\left(L_{i}\rho \left(\tau \right)L_{i}^{†}-\frac{1}{2}\left\{L_{i}^{†}L_{i},\rho \left(\tau \right)\right\}\right)$$

(5) $$J\;\left(\tau \right)=\;\mathrm{Tr}\left[j\left(\tau \right)\rho \left(\tau \right)\right]$$

where *H*(τ) is the Hubbard model Hamiltonian with the hopping parameter *t* replaced by $te^{-iaA_{\mathrm{pump}}\left(\tau \right)}$ and *a* is the lattice constant, i.e., the interatomic distance. ρ(τ) is the density operator in the real‐space basis and *L_i_* the Lindblad operator given by $L_{i}=n_{i}=c_{i↑}^{†}\;c_{i↑}+c_{i↓}^{†}c_{i↓}$ [25]. γ is the electron‐environment scattering rate. The current operator *j*(τ) is $j\;\left(\tau \right)=-ieat\sum_{\sigma }\sum_{i=1}^{N_{S}}\left(e^{-i\Phi (\tau )}c_{i\sigma }^{†}c_{i+1\sigma }-H.c.\right)$ with Φ (τ) = *aA*_pump_(τ). Starting from the ground state density of the Hubbard model, we solve the Lindblad equation using the fourth‐order Runge‐Kutta algorithm [44]. The Fourier transform of *J*(τ) would then result as a sum of harmonics of the incident optical pumping frequency, which would eventually emerge as the HHG spectrum *I*(ω), i.e., $I\left(\omega \right)\;∼\;\omega^{2}{\left|J(\omega )\right|}^{2}$. We evaluate the *n*th order harmonic intensity as $I_{n}=\int_{\left(n-0.5\right)\omega_{\mathrm{pump}}}^{\left(n+0.5\right)\omega_{\mathrm{pump}}}d\omega I\left(\omega \right)$, where ω_pump_ = 0.26 eV is adopted to be the pumping frequency in the present study. The vector potential could be written as $A_{\mathrm{pump}}\;\left(\tau \right)=\left(E_{\mathrm{pump}}^{0}/\omega_{\mathrm{pump}}\right)\;\sin\left(\omega_{\mathrm{pump}}\tau \right)\cos^{4}\left(\pi t/T_{\mathrm{env}}\right)$, where $E_{\mathrm{pump}}^{0}$ is the optical pump field strength. We take the pulse envelope period *T*_env_ to be 200 fs, where twelve optical cycles are enclosed in a pulse envelope. The pumping frequency is chosen to be in the mid‐infrared regime, where the driving cycle becomes comparable to the electron‐electron scattering time, allowing the electron‐electron scattering to actively participate in the electronic dynamics and leading to an abundant generation of hot carriers responsible for the broadband emission. Besides, in the study, we present calculations primarily for the system size of *N_S_* = 8, which would be acceptable for describing the local process, such as the doublon‐holon recombination. Actually, we have verified that the HHG spectra are qualitatively similar for *N_S_* = 6 and 8. Because the Lindblad equation with a dissipative quantum environment requires substantially higher computational costs (especially, memory) than TDSE, the system size is practically limited to *N_S_* = 8, corresponding to $14≲N_{S}≲16$ in TDSE in a respect of the computational cost. For a comparison between the Lindblad equation and QME, where the latter requires the Houston basis at each time step so that it demands more computational work, we restrict the system size to *N_S_* = 6 in QME of Figure 5.

### 4.2 Calculation of HHG with an Ad Hoc Extension of QME

QME could be written as the Lindblad equation with two Lindblad operators of *L*_1_ = σ_*x*_ − *i*σ_*y*_ and *L*_2_ = − σ_*z*_ in two‐level systems [11], which are well known to consider the density decay process via *T*_1_ and the spontaneous emission and the dephasing process via *T*_2_ in a phenomenological fashion. The two‐level Lindblad master equation (i.e., QME) can then be expressed as

(6) $$\frac{\partial }{\partial \tau }\rho \;\left(\tau \right)=-i\left[H\left(\tau \right),\rho \left(\tau \right)\right]+D\left[\rho \left(\tau \right)\right]$$

where ρ(τ) is the density operator and *D*[ρ(τ)] the relaxation operator. In the Houston basis [30], taking the simplest case of $T_{1}\to \infty$, the relaxation term *D*[ρ(τ)] can be represented as − (1 − δ_*mn*_)ρ_*mn*_(τ)/*T*_2_, where *m* and *n* are the indices of energy levels. This formulation may be extended on an ad hoc basis to multi‐level systems, for instance, to the Hubbard systems with multiple levels as large as the Hilbert space dimension, managing the Hubbard model, incorporating only a single relaxation channel with a dephasing time *T*_2_.

## Author Contributions

J.D.L. designed the study. G.B. performed analytic and numerical calculations. G.B., Y.K., and J.D.L. discussed the results and wrote the paper.

## Conflicts of Interest

The authors declare no conflicts of interest.

## Supporting information




**Supporting File**: advs74749‐sup‐0001‐SuppMat.docx.

## Data Availability

The data that support the findings of this study are available from the corresponding author upon reasonable request, and the numerical code used for the simulations is publicly available at https://github.com/giminbae/High‐Harmonic‐Generation‐Simulation‐Code.
