# Supplementary material for: Superradiance and Broadband Emission Driving Fast Electron Dephasing in Open Quantum Systems
Source: Adv Sci (Weinh). 2026 Mar 10;13(27):e22729. doi: 10.1002/advs.202522729 (PMC13170243; doi:10.1002/advs.202522729)
Supplement: Supplementary file 1 — Supporting File: advs74749‐sup‐0001‐SuppMat.docx. [file ADVS-13-e22729-s001.docx]

Supporting Information for

**Superradiance and Broadband Emission Driving Fast Electron Dephasing in Open Quantum Systems**

Gimin Bae^1^, Youngjae Kim^2^, and J. D. Lee^1*^

^1^*Department of Physics and Chemistry, DGIST, Daegu 42988, Republic of Korea*

^2^*School of Physics, KIAS, Seoul 02455, Republic of Korea*

^*^Correspondence and requests for materials should be addressed to J.D.L. ([jdlee@dgist.ac.kr](mailto:jdlee@dgist.ac.kr)).

**Supporting Information Text**

**I. Broadband emission and nonperturbative behavior**


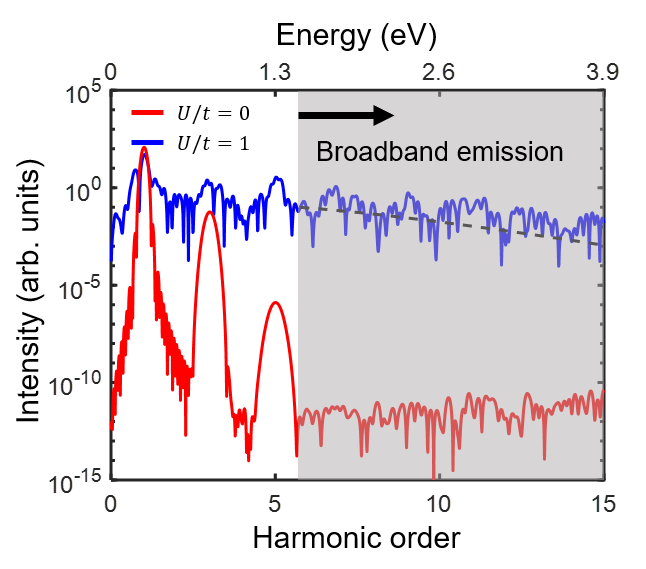


**Fig. S1.** High harmonic generation (HHG) and broadband emission in correlated electron systems. HHG spectra at $\gamma^{-1}=\infty$ fs for $U/t=0$ (red) and $U/t=1$ (blue) under the optical field strength 3.5 MV/cm. In this supporting information, the lattice constant is set to 4 Å and the hopping parameter to 0.52 eV, unless mentioned otherwise.

In Fig. S1, HHG spectra are delivered at $U/t=0$ and $1$. For $U/t=0$, only odd-order harmonics are generated. In contrast, at $U/t=1$, significant harmonics are observed regardless of whether they are odd or even order. This structureless emission can be evaluated as originating from excited carriers. This can be characterized as thermal-like radiation and fitted to blackbody emission intensity,

$$I_{B}\left( T_{\mathrm{eff}},\hbar\omega\right)\propto\frac{\omega^{3}}{\exp\left( \frac{\hbar\omega}{k_{B}T_{\mathrm{eff}}} \right)-1}, [S1]$$

where $T_{\mathrm{eff}}$ is the effective temperature, $\hbar$ Dirac constant, and $k_{B}$ Boltzmann constant. The black dashed line denotes the blackbody emission intensity $I_{B}\left( T_{\mathrm{eff}},\hbar\omega\right)$ at $T_{\mathrm{eff}}=3800$ K, which is fitted to the even-order harmonics. The gray shaded area represents energies from 1.5 to 4.0 eV, corresponding to the range where broadband emission of 2H-NbSe_2_ has been experimentally observed (1). It indicates that electron correlation is highly significant for broadband emission. Here the effective temperature extracted from the broadband emission should be understood as a transient quantity, defined immediately after the pumping, which parametrizes the energy scale and spectral distribution of the nonthermal carriers.


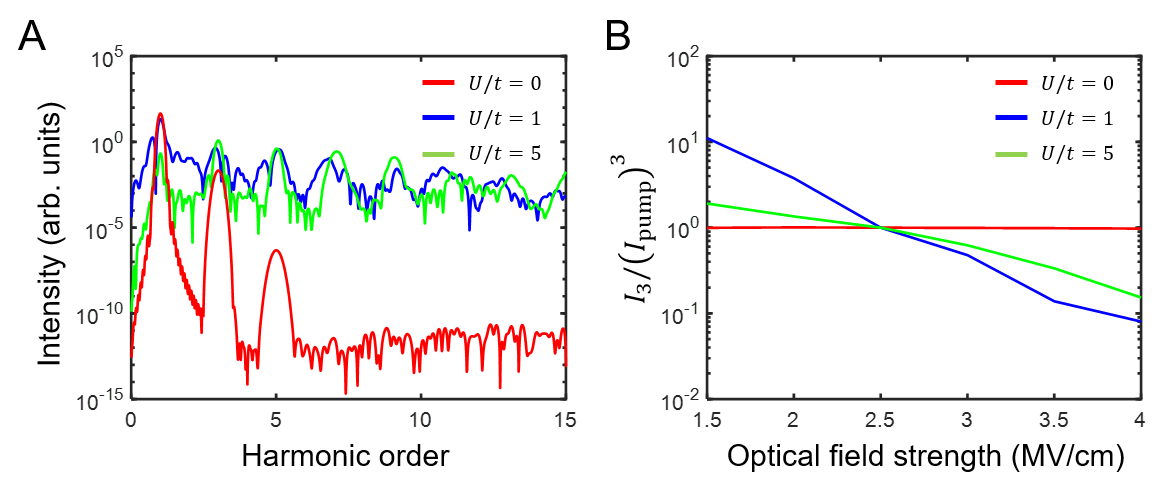


**Fig. S2.** HHG spectra of Hubbard chain with Lindblad master equation. (A) HHG spectra at $\gamma^{-1}=200$ fs for $U/t=0$, 1, and 5 under the optical field strength 3.5 MV/cm. (B) A ratio of third-order harmonic intensity and the cube of the optical pumping intensity, i.e., $I_{3}/I_{\mathrm{pump}}^{3}$, with respect to optical field strength.

In Fig. S2A, HHG spectra are delivered at $U/t=0$, 1 and $5$. Compared to the case without Lindblad coupling (Fig. S1 and Fig. 2B in main text), the noise is reduced, while the overall shape of the spectra remains qualitatively unchanged. Fig. S2B shows proper interplays between scattering and pumping processes intensify the nonperturbative behaviors of harmonics. This non-perturbative trend is reduced by the inclusion of superradiance, which is described through the Lindblad terms (2), compared to the case without it (see Fig. 2B in main text). This indicates that superradiance suppresses broadband emission, implying the presence of destructive interference between broadband emission and superradiance.

**II. HHG spectra for various conditions**


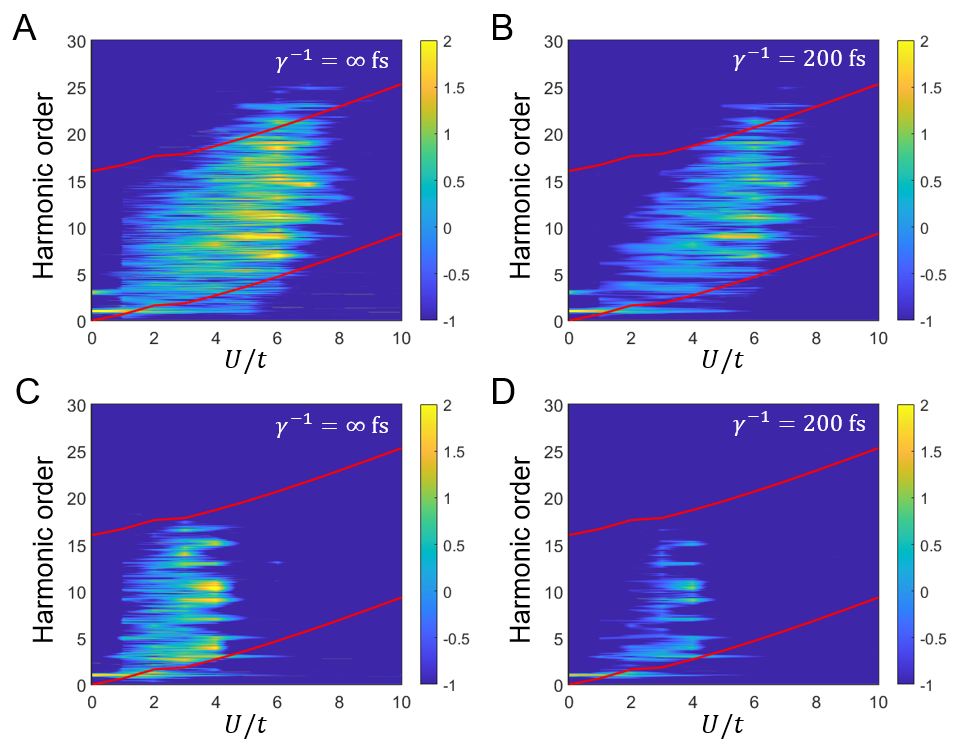


**Fig. S3.** HHG spectra with respect to $U/t$. (A and B) HHG spectra for $\gamma^{-1}=\infty$ fs (A) and $\gamma^{-1}=200$ fs (B) under the optical field strength 10 MV/cm. The red lines indicate $\Delta$ and $\Delta+8t$. (C and D) HHG spectra for $\gamma^{-1}=\infty$ fs (C) and $\gamma^{-1}=200$ fs (D) under the optical field strength 3 MV/cm.

Fig. S3 shows the HHG spectra for different conditions. Under a strong field (Fig. S3A), the insulator-metal phase transition enables the observation of harmonic spectra even in the insulating system ($U/t\gg1$) (3). The red lines indicate $\Delta$ and $\Delta+8t$, representing the energy range of the first allowed excitations in the half-filled system (3). The Lindblad coupling constant turns on, odd-order multiple harmonics become to develop from the structureless broadband emission (Fig. S3B). In the weak field regime, the range of the insulator-metal phase transition narrows, resulting in strong harmonic spectra under a weak or moderate electron correlation ($1\lesssim U/t\lesssim3$). At higher harmonic orders, the main spectral peak is positioned near the doublon excitation energy, $U/\omega_{\mathrm{pump}}$.

**III. Correlations and superradiance**


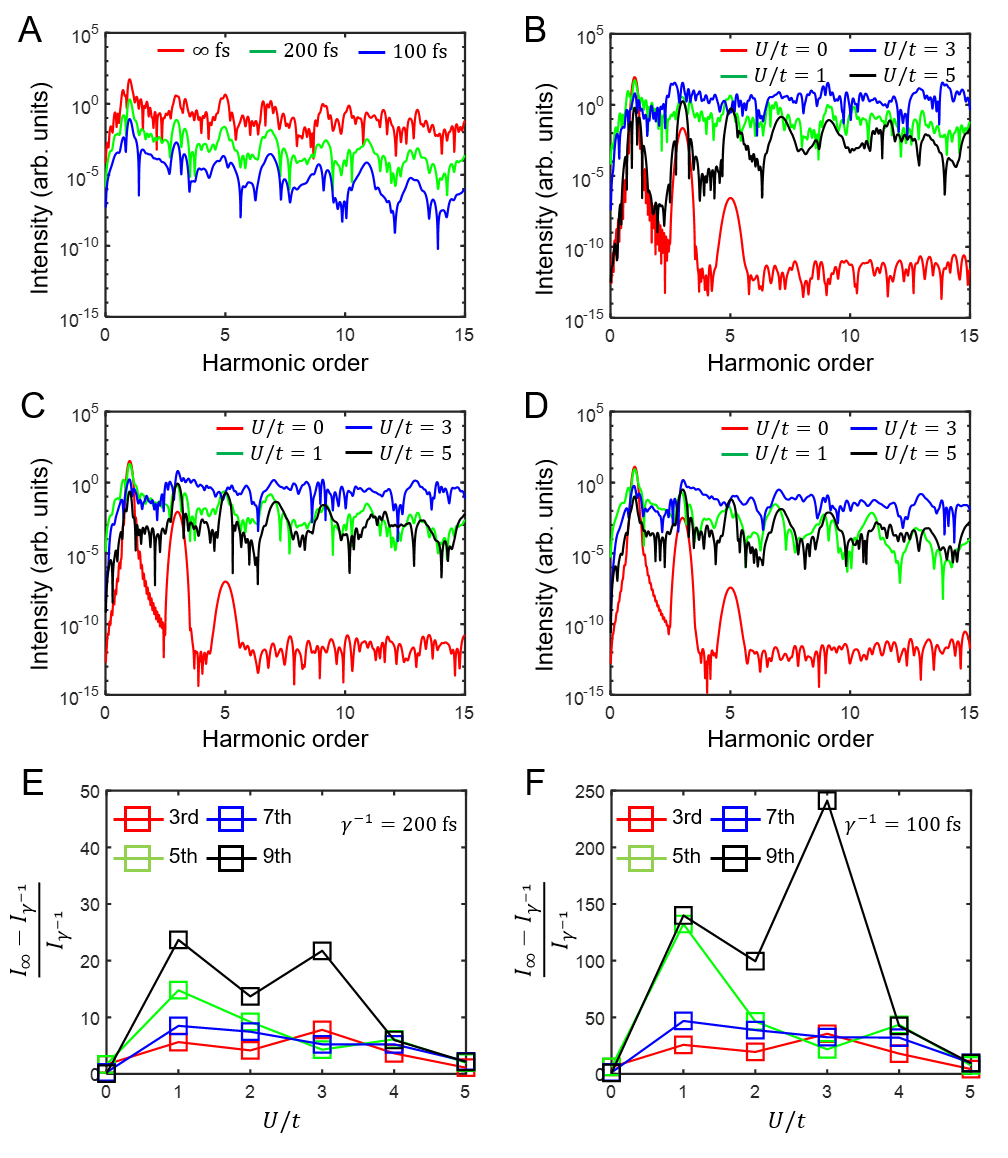


**Fig. S4.** HHG spectra of Hubbard chain: Dicke superradiance and broadband emission. (A) HHG spectra at $U/t=1$ for $\gamma^{-1}=\infty, 200, 100$ fs under the optical field strength 3 MV/cm. For a better demonstration of the spectral shapes, spectra are vertically offset by multiplying factors of $1, {10}^{-1}, {10}^{-2}$. (B) HHG spectra for $\gamma^{-1}=\infty$ fs under the optical field strength 3 MV/cm with respect to $U/t$. (C) HHG spectra for $\gamma^{-1}=200$ fs under the optical field strength 3 MV/cm with respect to $U/t$. (D) HHG spectra for $\gamma^{-1}=100$ fs under the optical field strength 3 MV/cm with respect to $U/t$. (E and F) Normalized differences between harmonic strengths at $\gamma^{-1}=\infty$ and $\gamma^{-1}=200$ fs (E) and $\gamma^{-1}=100$ fs (F) for the third, fifth, seventh, and ninth order harmonics with respect to $U/t$.

In Fig. S4A, HHG spectra are evaluated at fixed values of $U/t=1$ with respect to $\gamma^{-1}=\infty, 200,$ and $100$ fs. In Fig. S4B-D, HHG spectra are displayed at $\gamma^{-1}=\infty, 200,$ and $100$ fs, respectively, at $U/t=0, 1, 3,$ and $5$. As the value of $U/t$ deviates from the weakly or moderately correlated regime ($U/t\to0$ or $U/t\to\infty$), the structure of odd-order harmonics becomes more pronounced in each figure. Further, it is noticeable that absolute strengths of harmonic spectra monotonically decrease as $\gamma^{-1}$ decreases order by order at all values of given $U/t$ (similar to Fig. 3 in main text). For the third, fifth, seventh, and ninth order harmonics, the normalized differences between those absolute strengths at $\gamma^{-1}=\infty$ and $\gamma^{-1}=200$ fs are investigated in Fig. S4E and the normalized differences between at $\gamma^{-1}=\infty$ and $100$ fs in Fig. S4F with respect to the $U/t$, which are discovered to dramatically increase as $U/t$ approaches the range of weakly or moderately correlated regime ($1\lesssim U/t\lesssim3$). Moreover, the normalized difference at $\gamma^{-1}=100$ fs shows more prominent increase that that at $\gamma^{-1}=200$ fs. This suggests that the electron correlation $U$, as well as the interatomic distance $a$ is a key parameter in inducing Dicke superradiance (Fig. 3 in main text).

It can be shown that the normalized difference ($I_{\infty}-I_{\gamma^{-1}})/I_{\gamma^{-1}}$ approximates the Dicke superradiance in the limit of a small interatomic distance (lattice constant). One may put $I_{\infty}=I_{0}+I_{B}$, $I_{\gamma^{-1}}=I_{0}+\left| A_{B}-A_{S} \right|^{2}\approx I_{0}+I_{B}-2A_{B}A_{S}$, and $I_{\infty}-I_{\gamma^{-1}}\approx2A_{B}A_{S}$, where $I_{B}\gg I_{S}$ is assumed and, for the simplicity, the amplitudes are taken to be real, i.e., $I_{B}=A_{B}^{2}$ (broadband emission) and $I_{S}=A_{S}^{2}$ (superradiance). We note $1/{I_{\gamma^{-1}}}=1/{{(I}_{0}+I_{B}-2A_{B}A_{S}})\approx1/{{(I}_{0}+I_{B})}+{2A_{B}A_{S}}/{{{(I}_{0}+I_{B})}^{2}}$, where the first term is independent of the lattice constant and the second term could be dominant in the limit of a small lattice constant. Therefore, we can get ($I_{\infty}-I_{\gamma^{-1}})/I_{\gamma^{-1}}\propto I_{S}$, which could approximate the Dicke superradiance in the destructive interference between the broadband emission and the Dicke superradiance in the limit of a small interatomic distance.

Here it is expected that, in the limit of $U/t\to0$, the Dicke superradiance becomes totally absent because a vanishing of the correlation should suppress the many-body dephasing and deexcitation, whereas, in the opposite limit of $U/{t\gg1}$, the system becomes insulating and the electron excitation (creation of doublon-holon pairs) is suppressed, which also quenches a channel of the Dicke superradiance. This is consistent with Fig. S4E-F.

**IV. Steady state and Lindblad operators**


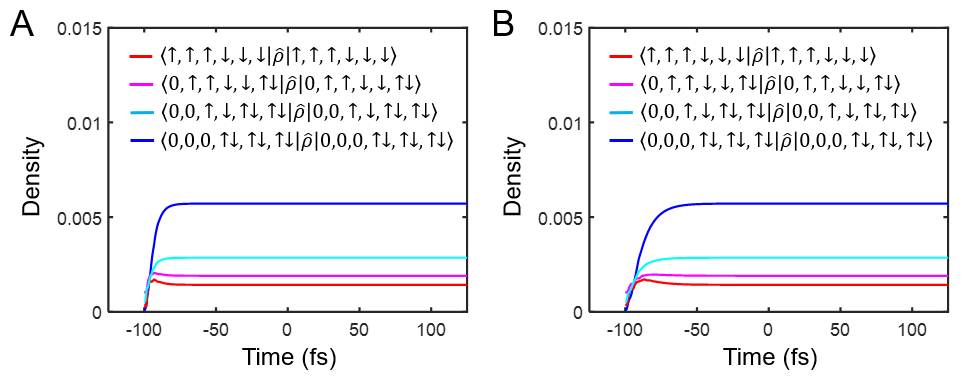


**Fig. S5.** Density calculations with Lindblad master equation. (A) Densities of subset of many-body states obtained by the Lindblad master equation in the one-dimensional Hubbard model $(N_{S}=6)$ at $U/t=1$ for $\gamma^{-1}=4 \mathrm{fs}$ under the optical field strength $3 MV/cm$. The Lindblad operator $L_{i}$ is given by the number operator $L_{i}=n_{i}=c_{i\uparrow}^{\dagger}c_{i\uparrow}+c_{i\downarrow}^{\dagger}c_{i\downarrow}$. The subset is selected based on the number of doublons. For example, state $\left| \uparrow,\uparrow,\uparrow,\downarrow,\downarrow,\downarrow\right\rangle$ has no doublons. (B) Densities of subset of many-body states with the different Lindblad operator $L_{i}=n_{i\uparrow}n_{i\downarrow}$. Same parameters are adopted as in (A).

We calculate the densities of subset of many-body states with Lindblad master equation. In Fig. S5, each figure adopts a different Lindblad operator. In the case of the Lindblad operator $L_{i}=n_{i}$ (Fig. S5A), it converged to a steady state characterized by $\rho_{D=0}:\rho_{D=1}:\rho_{D=2}:\rho_{D=3}=\frac{1}{4}:\frac{1}{3}:\frac{1}{2}:1$, where $D$ is the number of doublons. Interestingly, in the case (4) of the Lindblad operator $L_{i}=n_{i\uparrow}n_{i\downarrow}$ (Fig. S5B), it converged to the same steady state. Due to electron-environment scattering, these cases ultimately converge to a specific steady state. As a result, qualitatively similar outcomes are expected regardless of the choice of the Lindblad operator.


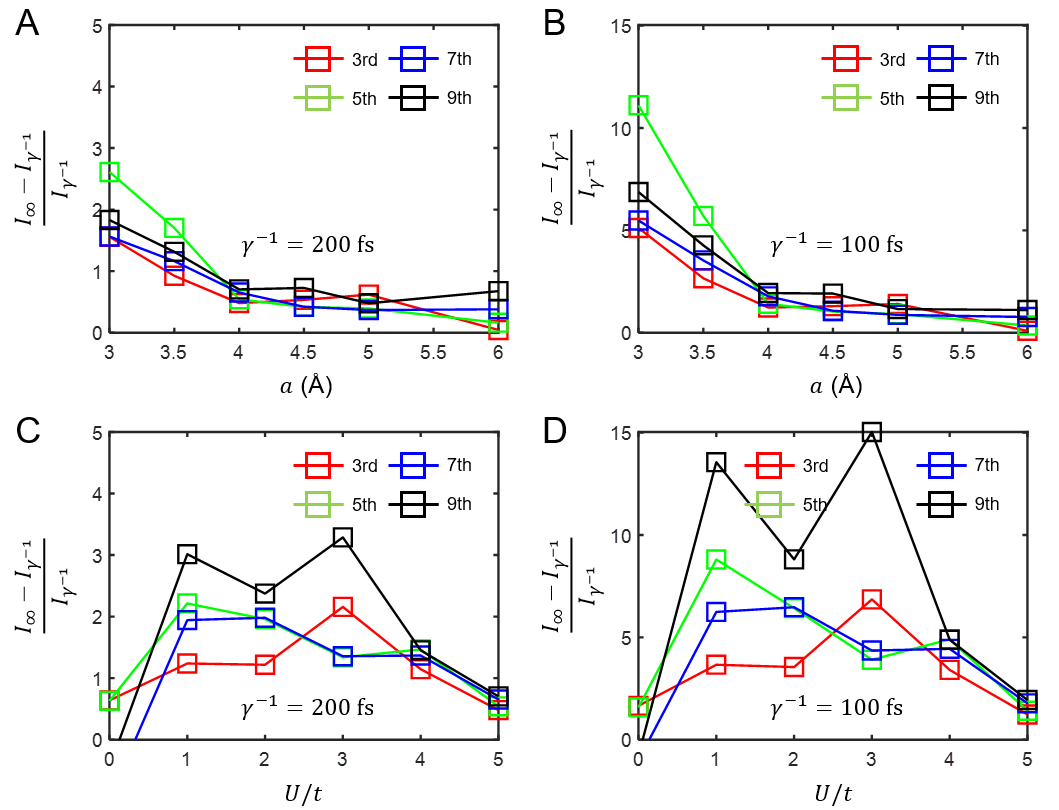


**Fig. S6.** Dicke superradiance and broadband emission with Lindblad operator $L_{i}=n_{i\uparrow}n_{i\downarrow}$. (A and B) Normalized differences between harmonic strengths at $\gamma^{-1}=\infty$ and $\gamma^{-1}=200$ fs (A) and $\gamma^{-1}=100$ fs (B) for the third, fifth, seventh, and ninth order harmonics with respect to $a$. (C and D) Normalized differences between harmonic strengths at $\gamma^{-1}=\infty$ and $\gamma^{-1}=200$ fs (C) and $\gamma^{-1}=100$ fs (D) for the third, fifth, seventh, and ninth order harmonics with respect to $U/t$.

We performed the calculations by applying the Lindblad operator $L_{i}$ as $n_{i\uparrow}n_{i\downarrow}$. For the third, fifth, seventh, and ninth order harmonics, the normalized differences between those absolute strengths at $\gamma^{-1}=\infty$ and $\gamma^{-1}=200$ fs are investigated in Fig. S6A and the differences between at $\gamma^{-1}=\infty$ and $100$ fs in Fig. S6B with respect to the $a$, which are discovered to dramatically increase as $a$ decreases (Fig. 3 in main text). Moreover, the normalized difference at $\gamma^{-1}=100$ fs shows more prominent increase that that at $\gamma^{-1}=200$ fs. In Figs. S6C and D, a similar trend is observed in the calculations with respect to $U/t$, which qualitatively corresponds to Figs. S4E and F. These results suggest that the same conclusion can be drawn regardless of the detailed form of the spin-independent Lindblad operator.

**References**

1. K. S. Takeda, et al. Ultrafast Electron-Electron Scattering in Metallic Phase of 2H-NbSe_2_ Probed by High Harmonic Generation. *Phys. Rev. Lett.* **132**, 186901 (2024).

2. S. Sarkar, S. Langer, J. Schachenmayer, A. J. Daley, Light scattering and dissipative dynamics of many fermionic atoms in an optical lattice. *Phys. Rev. A* **90**, 023618 (2014).

3. R. E. F. Silva, I. V. Blinov, A. N. Rubtsov, O. Smirnova, M. Ivanov, High-harmonic spectroscopy of ultrafast many-body dynamics in strongly correlated systems. *Nat. Photonics* **12**, 266-270 (2018).

4. J.-S. Bernier, D. Poletti, C. Kollath, Dissipative quantum dynamics of fermions in optical lattices: A slave-spin approach. *Phys. Rev. B* **90**, 205125 (2014).
